# Efficacy of Xpert MTB/RIF assay in detecting *Mycobacterium tuberculosis* in samples with different results by smear and culture in a coastal city with high incidence of tuberculosis

**DOI:** 10.1186/s12879-025-10446-z

**Published:** 2025-01-11

**Authors:** Gang Feng, Hongyu Jiang, Ying Chen

**Affiliations:** 1 School of Medical Technology, Xuzhou Medical University, Xuzhou, 221004 China; 2 The Fourth People’s Hospital of Lianyungang City, Lianyungang, Jiangsu Province, China

**Keywords:** Tuberculosis, Drug resistance, Xpert MTB/RIF, Diagnosis

## Abstract

**Background:**

Tuberculosis (TB) is a global problem that seriously jeopardizes human health. Among them, the diagnosis and treatment of smear- or culture-negative TB patients is a challenge. The Xpert MTB/RIF (Xpert) assay has been reported to be a novel molecular diagnostic tool for rapidly detecting TB. Still, there is limited data on this assay's performance in subgroups of TB patients. This study aimed to evaluate the diagnostic value of the Xpert method in patients with different smear and culture results and to assess its efficacy for rifampicin resistance (RR) detection.

**Methods:**

We retrospectively collected data from 1,721 patients with a clinical diagnosis of tuberculosis. Smear, Xpert, and traditional solid culture methods were used to detect TB infection and explore the detection rate of Xpert in the grouping of results from different smear and culture methods. Information on RR detected by the Xpert method and proportional method of drug sensitivity test (DST) was also recorded and kappa values, sensitivity, and specificity were calculated.

**Results:**

We observed that among the three methods, the Xpert method had the highest detection rate of 66.8%, followed by the culture method at 56.0% and the smear method had the lowest at 40.0%. The detection rate of Xpert was 98.3% (642/653) when both smear and culture were positive, 85.1% (296/348) when only one of the two methods, smear and culture, was positive, and 29.4% (212/720) when both smear and culture were negative. The Xpert method and DST showed a high agreement (κ = 0.92) for RR detection. The highest mutation rate was observed for probe E (64.7%), and the least number of probe C mutations occurred (1.5%).

**Conclusion:**

The Xpert method has high detection efficiency. It has good diagnostic value in detecting *MTB* and RR, especially in cases where traditional culture and sputum smear results are negative, and significantly reduces the rate of missed diagnosis.

## Background

Tuberculosis (TB) is a serious global public health problem caused by *Mycobacterium tuberculosis* (*MTB*), which is also one of the ten leading causes of human mortality [1]. Since the introduction of the rifampicin-based treatment regimen in clinical practice in 1972, standardized treatment regimens have been used for over half a century. However, the occurrence and development of TB have not been fully contained. To this day, it remains one of the three most serious infectious diseases that threaten human health, along with AIDS and malaria. It threatens the health and safety of nearly one-third of the world's population [2]. The number of detected and reported cases of TB increased from 9.96 million in 2019 to 10.8 million in 2023. Of these, 55% were adult males, 33% were adult females, and 12% were children and adolescents, of whom only 48% of patients diagnosed with TB were initially tested using the rapid test recommended by the World Health Organization (WHO) [3]. Over 90% of new cases and deaths are in developing countries, with India, Indonesia, and China accounting for the most cases. In addition, with the widespread spread of TB, the problem of *MTB* drug resistance is becoming more and more serious [4, 5]. The WHO has proposed a plan to eliminate TB by 2035. It is particularly urgent to promote rapid and accurate diagnostic methods [6].

Most TB-endemic countries and regions still rely on acid-fast bacilli (AFB) staining-based microscopy and culture methods. Ziehl–Neelsen (ZN) smear microscopy is widely used due to its ease of use and low cost, however, TB transmission due to missed TB cases from negative smears has become a global health problem [7]. The sensitivity of the ZN smear method is low, ranging from 34 to 80%, and the lower the *MTB* load in sputum, the lower the detection rate [8, 9]. Additionally, it cannot distinguish between *MTB* and *nontuberculous mycobacteria* (*NTM*). Meanwhile, it has been reported that there are risks associated with the preparation of TB sputum smears, including aerosol generation and inhalation [10, 11]. Although the results of the traditional Lowenstein-Jensen (L-J) culture method and drug sensitization tests (DST) are reliable, it takes weeks to obtain the results, which may lead to delays in the treatment and diagnosis of patients and increase their suffering and burden. Additionally, the culture method requires complex biosafety equipment and cumbersome experimental procedures, which hinders the popularization and application of the technique [10, 12]. Also, the presence of other bacteria in the sample, low *MTB* load in the specimen, and operator error in the experimental procedure resulted in inconclusive or negative culture results, resulting in missing data [13–15].

The diagnosis of TB cases with negative smear results is challenging. It is clinically difficult to provide an accurate diagnosis and chemotherapy regimen promptly for patients with symptoms of TB. Still, negative smear results, and in most cases, such patients can be treated in a standardized and effective manner only after definitive culture results are obtained [16]. Although patients with low loads are less infectious and have lower mortality rates relative to AFB-positive patients, studies have shown that 50.0–71.0% of patients develop active TB, with a similarly high proportion of drug-resistant patients in this group [17, 18]. Relative to AFB-positive patients, 90% of AFB-negative patients require longer chemotherapy dosing [19]. False-negative results lead to patients not being properly diagnosed and treated promptly, leading to increased mortality [20], especially when the infecting strain is a drug-resistant strain. Underdiagnosis due to sputum smear negativity is a global problem. Previous studies have shown that about 12.6% of TB patients have negative sputum smears but positive culture results [21]. This demonstrates the need for more sensitive, accurate, and less time-consuming diagnostic tools to facilitate timely and accurate clinical diagnosis to reduce the rate of missed and misdiagnosed cases, minimize patient suffering and economic burden, and reduce the risk of transmission and mortality within the population. Rapid and accurate diagnosis of TB is essential for TB prevention and control, as well as for combating the spread of drug-resistant TB.

**Fig. 1:**
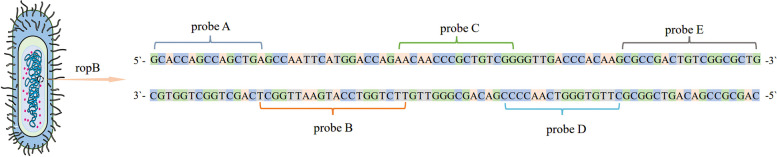
Sequences of the *rpoB* gene corresponding to the five probes

In recent years, the WHO has recommended the Xpert MTB/RIF (Xpert) test as a preliminary diagnostic test for individuals suspected of having TB in countries where the disease is endemic. The method uses an in vitro diagnostic reagent and a nested real-time fluorescence quantitative PCR method that simultaneously detects *MTB* complex DNA and RR-associated *rpoB* mutations in sputum samples. It has high sensitivity and specificity for the detection of *MTB*. The detection limit of the Xpert method was evaluated to be 136 bacilli/ml [10]. Compared to traditional methods, the Xpert method requires less laboratory biosafety, is easy to automate, takes only 2 h to perform, and is less susceptible to cross-contamination [22, 23]. Previous studies have shown that up to 95% of RR is associated with mutations in the 81 bp region of the *rpoB* gene [24]. The Xpert assay uses five overlapping probes in the kit for the *rpoB* gene: probe A (codons 507–511), probe B (codons 511–518), probe C (codons 518–523), probe D (codon 523–529) and probe E (codon 529–533) (Fig. 1). A positive specimen that is negative for one or more probes indicates that the sample is resistant to rifampicin.

Studies on the performance of Xpert in sputum AFB-negative and culture-positive or both AFB and culture-negative samples from clinically diagnosed cases are scarce and involve small sample sizes. To date, there has been no assessment of the accuracy of the Xpert method for detecting *MTB* and RR in samples with different AFB grades and culture results. Therefore, this study aimed to determine the diagnostic accuracy of the Xpert method for detecting *MTB* and RR in pulmonary samples of various AFB smear grades and culture results.

## Materials and methods

### Case inclusion and subgrouping

Lianyungang City is a coastal city in eastern China and northern Jiangsu Province, with an area of 7,615 square kilometers. It has four counties and two districts under its jurisdiction, with a resident population of 4.59 million. Lianyungang is the city with the lowest economic level and the lowest per capita GDP in Jiangsu Province. The Fourth People's Hospital of Lianyungang is the only local municipal public infectious disease hospital responsible for TB screening and treatment. The present study retrospectively evaluated the laboratory results and clinical data of TB patients and patients with suspected TB who were seen at the Fourth People's Hospital of Lianyungang from June 2020 to December 2023. A total of 2,329 patients who underwent the smear method, TB culture, and Xpert testing were included in this study. Patients were categorized into two groups based on their final clinical diagnosis: the TB group and the non-TB group. The latter group includes individuals with a definitive diagnosis of lung disease unrelated to *MTB*, such as acute and chronic pneumonia, lung cancer, pulmonary nodular disease, and *NTM* infections.

The diagnostic criteria are as follows:

1. A history of tuberculosis.
2. Clinical signs and symptoms of tuberculosis.
3. Radiologic and imaging manifestations consistent with tuberculosis.
4. Positive laboratory pathologic tests (antacid bacillus smear, *MTB* nucleic acid test, *MTB* culture), or typical lesions on pathologic examination.
5. Positive immunologic tests, such as tuberculin skin or interferon release tests.

Classification as TB requires a combination of any of the following criteria: (2) and (4); (1), (2), and (5); (1), (3), and (5); or (2), (3), and (5), as well as a patient who is on effective diagnostic anti-tuberculosis therapy [25].

### Specimen collection

Subjects were asked to provide one cup of sputum immediately, one cup at bedtime, and three cups in the morning. Before collecting the sputum specimens, subjects should be calm, rinse their mouths with water, take a deep breath, and cough up sputum from deep in the lungs. The sputum should then be collected into special sterile sample cups, with each cup containing no less than 2 ml of sputum. One cup of morning sputum was used for the Xpert assay and one cup of morning sputum was used for culture. The remaining three cups of sputum were stained with ZN stain and examined microscopically. Bronchoalveolar lavage fluid (BALF) was collected by a clinician via bronchoscopy and dispensed into three special sterile sample cups for each method.

### Acid-fast bacilli staining and microscopic examination

**Fig. 2:**
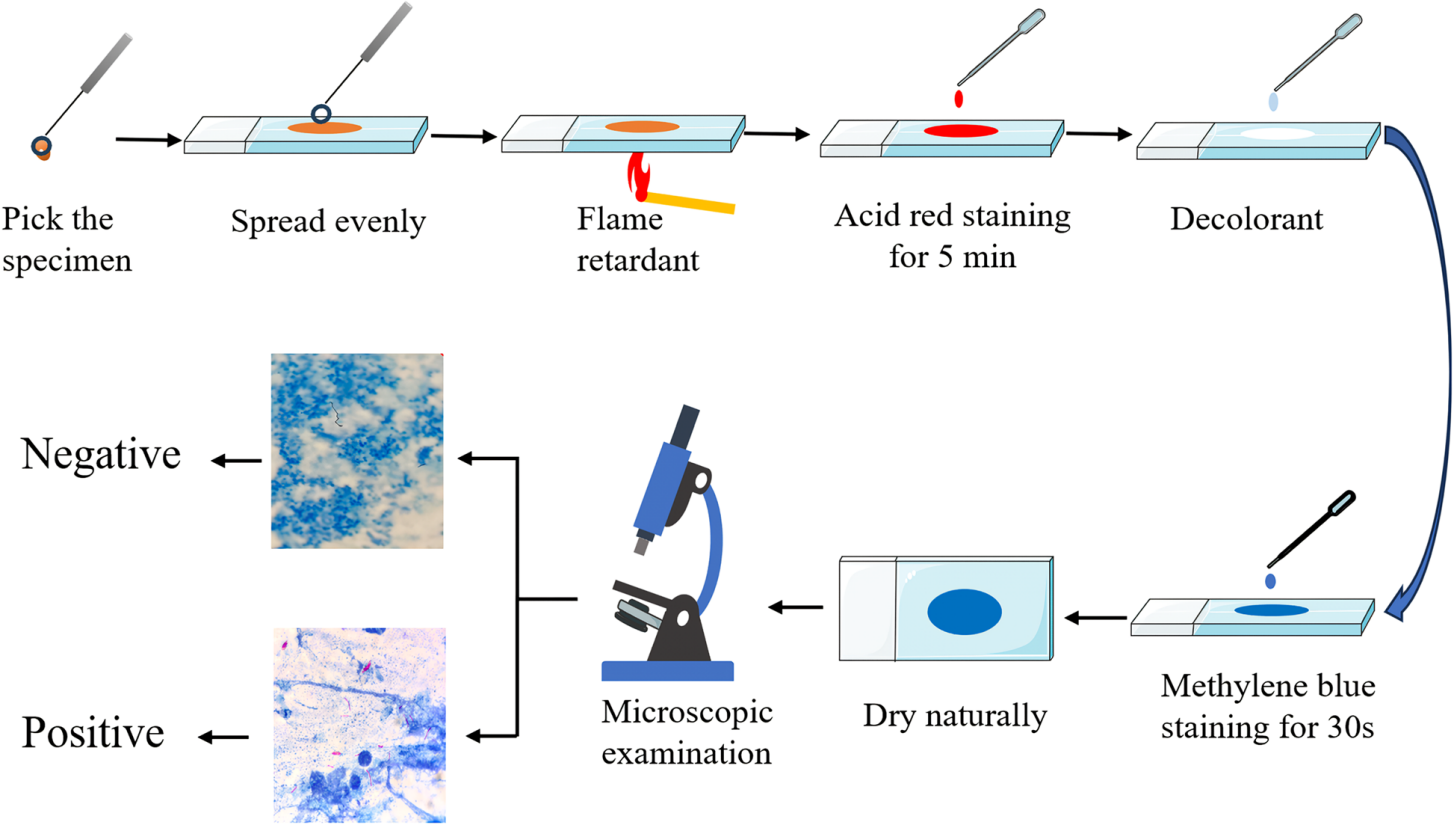
Flow chart of the smear antacid staining assay

Pick 0.05 ml of the suspected specimen component, and spread an ovoid film evenly on the slide. Allow it to dry naturally and fix it by passing it over a flame before staining. Staining was performed using Baso (Zhuhai, China) acid-fast staining solution (Ziehl–Neelsen method), and the staining process is shown in Fig. 2. The stained smears were placed under a microscope and observed using an oil immersion lens (1,000x). The results were reported in six forms. (1) Negative: no acid-fast bacilli were found in 300 visual fields. (2) AFB Trace: 1–8 bacilli in 300 visual fields. (3) AFB 1 + : 3–9 bacilli in 100 visual fields. (4) AFB 2 + : 1–9 bacilli in 10 visual fields. (5) AFB 3 + : 1–9 bacilli per visual field. (6) AFB 4 + : 10 or more bacilli per visual field.

### MTB culture and the proportional method of DST

**Fig. 3:**
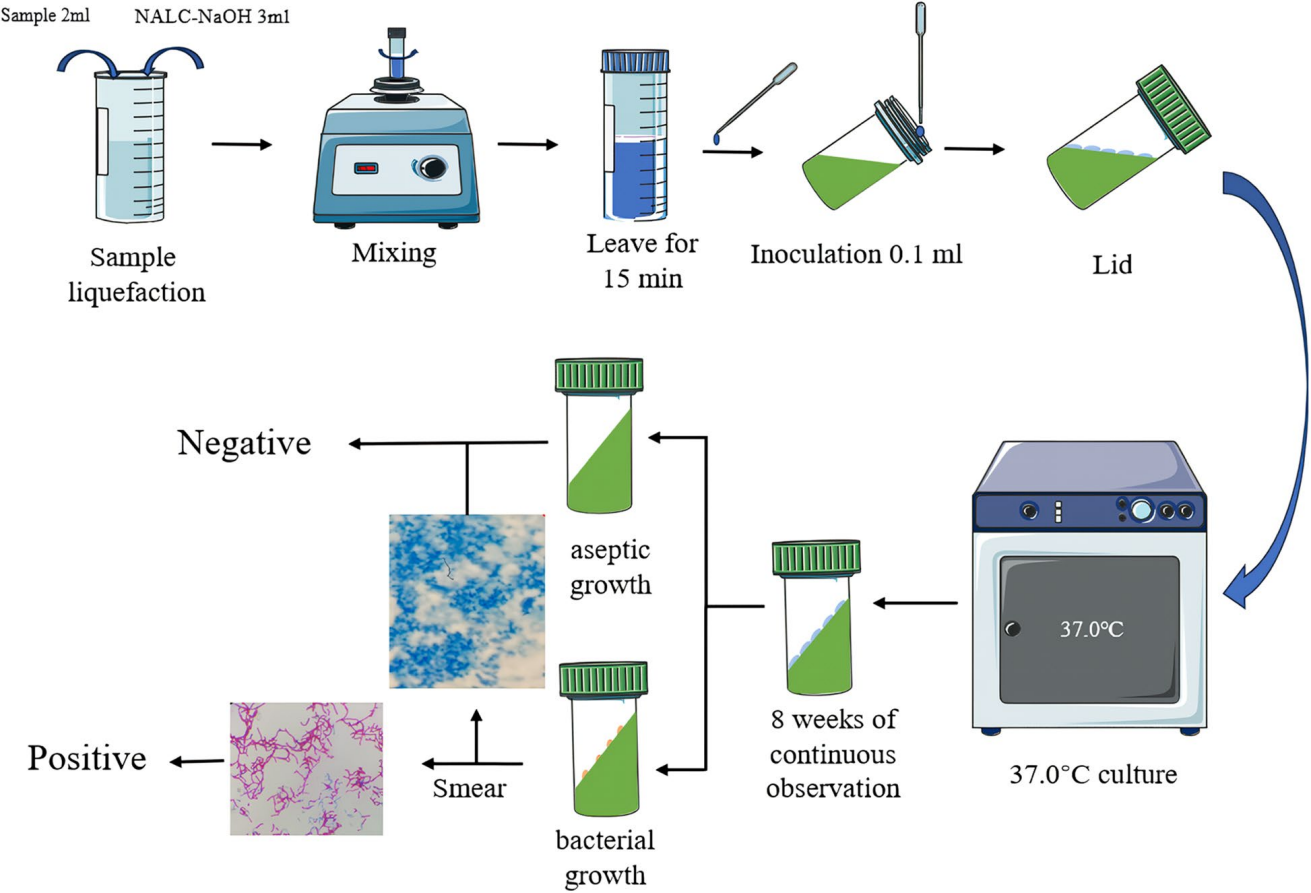
Flow chart of traditional culture by solid method

Sputum or BALF must be pre-treated before culture by adding the specimen and N-acetyl-L-cysteine-NaOH (NALC-NaOH) digest at a ratio of 1:1.5 to a large centrifuge tube, mixing with sufficient shaking, and then allowing to stand for 15 min. A single-use sterile pipette was used to aspirate 100 μl of digested specimens and inoculate them into 2 sticks of L-J Solid Medium (Baso, Zhuhai, China). The acidic component of the medium neutralizes the alkalinity in the specimen. The medium was placed in a 37 ℃ incubator and the results were observed once a week for 4–8 weeks. Those who had not found any colonies visible to the naked eye after 8 weeks were considered negative. In the medium with colony growth, smear antacid staining of the L-J culture, positive staining was reported as “culture-positive”, and negative staining was reported as “culture-negative” (Fig. 3). Culture-positive strains will be used for further DST testing.

**Fig. 4:**
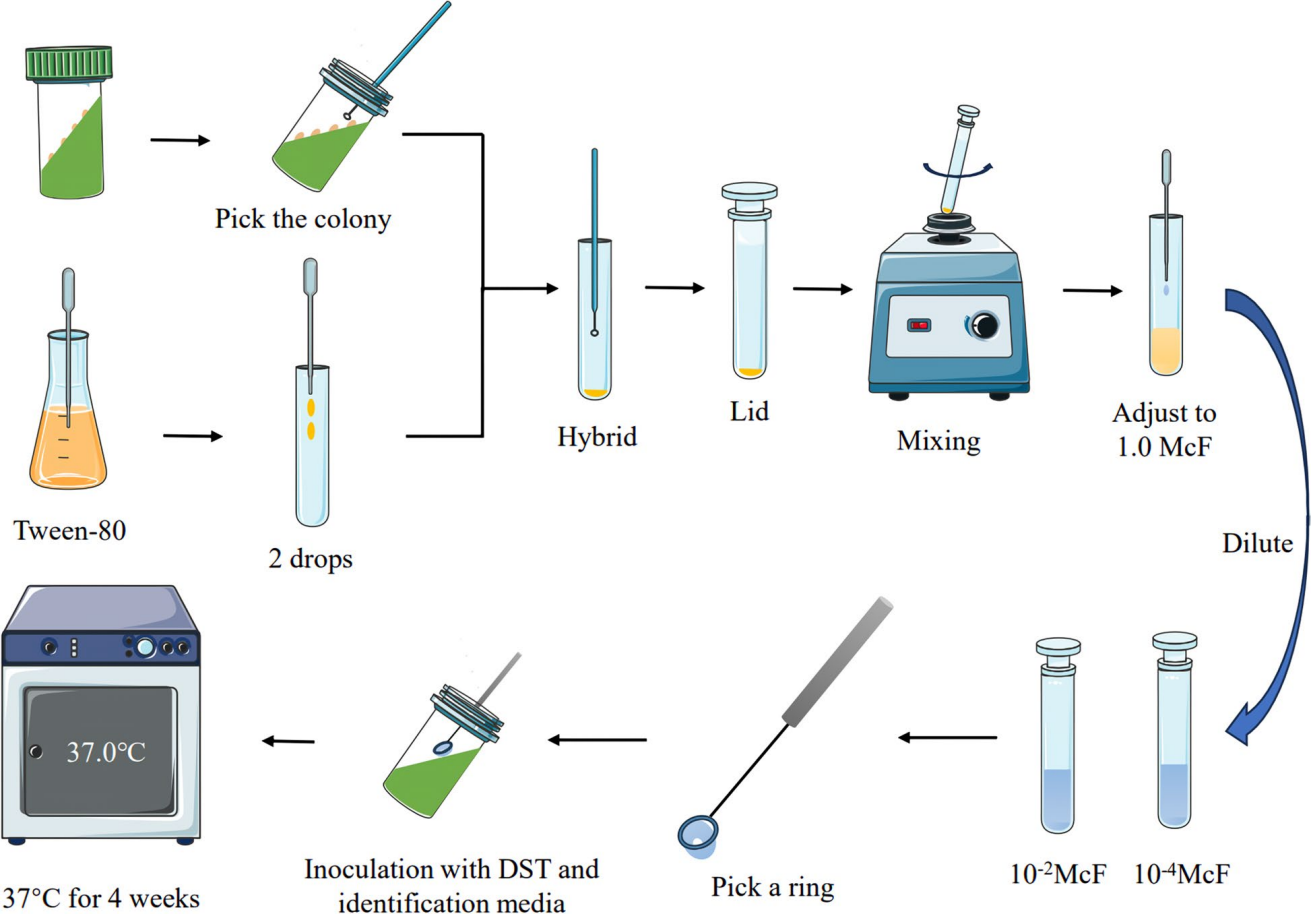
Flow chart of the proportion method of drug sensitization test. McF: McFarland

Pick the culture-positive strains and grind them into 1.0 McF of bacterial suspension, then dilute the suspension to 10^–2^ McF and 10^–4^ McF sequentially, and pick 10 µl (i.e., one ring) of the suspension with 22 SWG standard inoculation ring and inoculate it into the medium containing rifampicin, and use the same method to inoculate the identification medium containing p-nitrobenzoic acid and the medium of the control group which doesn't contain the drug. The results were observed after 4 weeks of continuous incubation in a 37℃ incubator (Fig. 4), and the number of colonies on the medium was counted and the resistance rate was calculated. The experiment failed if no colony growth was seen on the control medium. If the control medium was positive and the identification medium was negative, the identification result was *MTB*, if both the identification medium and the control medium were positive, it meant the growth of *NTM*. Resistance rate (%) = (number of colonies grown on drug-containing medium/number of colonies grown on control medium) × 100%. Sensitive: resistance rate < 1%, resistant: resistance rate > 1%.

### Xpert for detection of MTB and RR

**Fig. 5:**
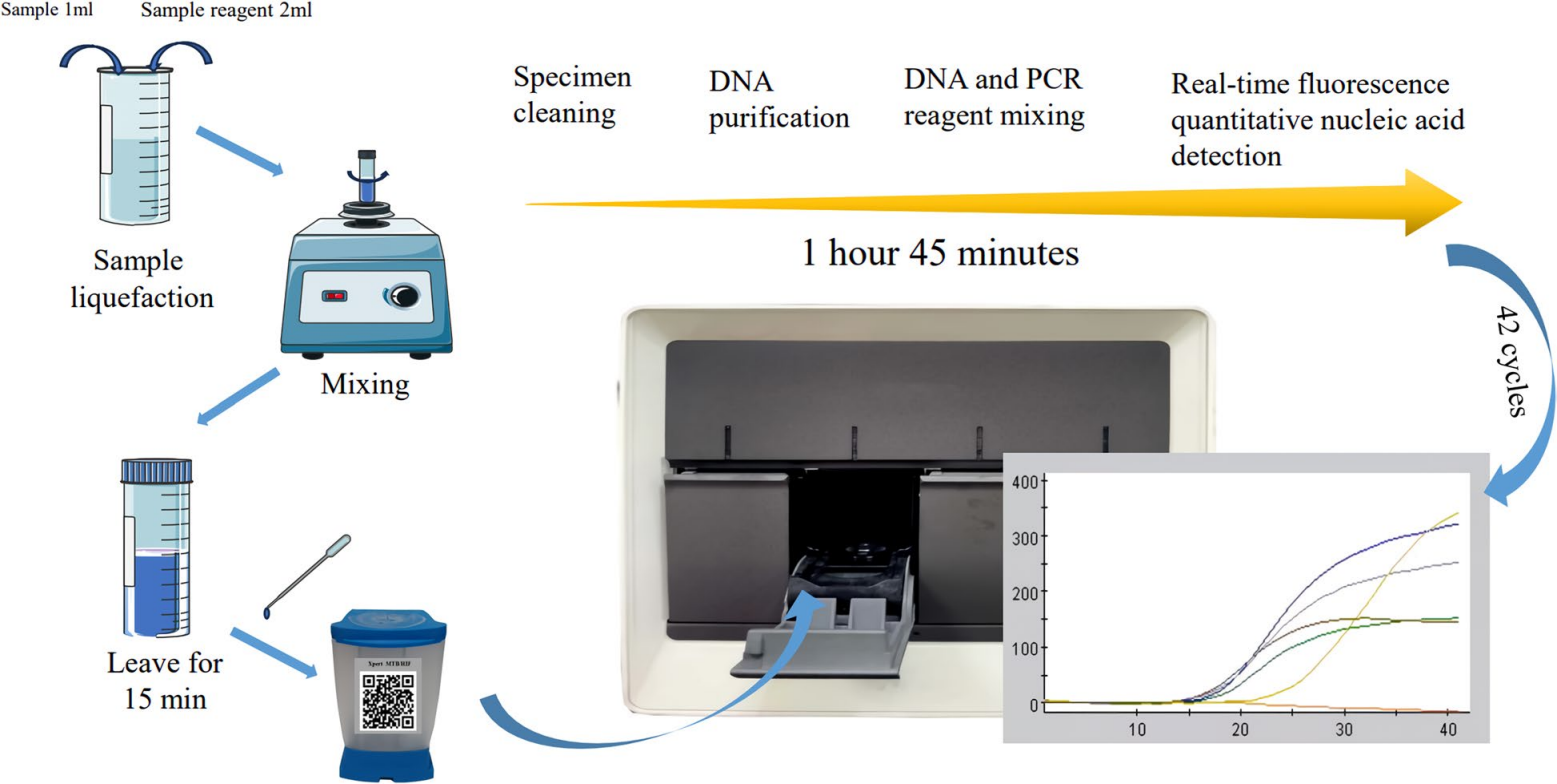
Flow chart of the Xpert method experiment

Take 1.0 ml of sample, add 2.0 ml of sample reagent, fully vortex and oscillate, then leave at room temperature for 15 min. 2.0 ml of the sample digest to be tested was added to the reagent cartridge (Cepheid, USA) and put into the test chamber, and then the test result was read out in the window of the system testing software after 105 min (Fig. 5). Tuberculosis test results were reported as “*MTB* not detected” or “*MTB* detected” (very low, low, medium, high).

**Fig. 6:**
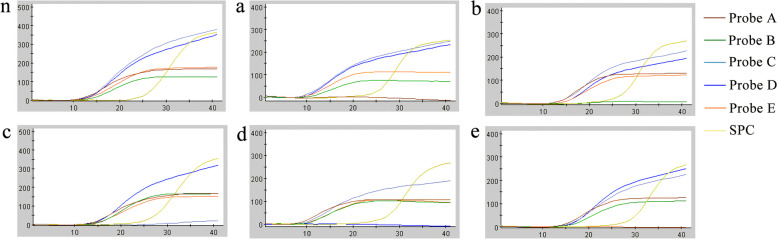
Pattern map of several common drug resistance gene mutations detected by the Xpert method. SPC: Sample processing quality control; (n) Rifampicin resistance was not detected; a to e in the figure denotes probe A to probe E mutations, respectively

Rifampicin resistance test results were displayed only if *MTB* is detected, and the results were reported in three forms. (i) Rifampicin resistance detected: a mutation in the *rpoB* gene was detected and the result was within the validated delta Ct set value. (ii) Indeterminate: the concentration of *MTB* was so low that resistance cannot be determined. (iii) Rifampicin resistance was not detected: no *rpoB* gene mutation was detected (Fig. 6).

### Statistical analysis

The data collected were entered into Excel sheets and independently checked by at least two researchers to confirm the accuracy of the data in the Excel sheets. The data were initially organized using Excel 2016 (Microsoft Corporation). The results of the study were mainly presented in the form of tables and charts. Data were expressed as frequencies and percentages. The detection rates of the various methods were evaluated using clinical diagnosis as a reference, receiver operating characteristic curves (ROC) were plotted and the area under the curve (AUC) was calculated. DeLong's test was used to compare the differences between the ROC curves, with *P* < 0.05 indicating a statistically significant difference. The sensitivity, specificity, positive predictive value (PPV), and negative predictive value (NPV) of the Xpert methods were evaluated for different smear results using L-J culture results as the reference standard. The degree of agreement between the DST and Xpert for RR detection was also assessed using Cohen's kappa coefficient (ĸ). ĸ values greater than 0.75 indicate very good agreement between the two methods, whereas an ĸ value of 0.4 to 0.75 indicates fair to good agreement between the two methods. A value of ĸ less than 0.4 indicates poor agreement between the two methods. AUCs were compared in the MedCalc 19.5.6 (Mariakerke, Belgium) software. Data were analyzed using SPSS 21.0 (Statistical Package for the Social Sciences, Inc., Chicago, Illinois, USA) and GraphPad Prism 8 (San Diego, CA, USA) software.

## Results

### Sociodemographic and clinical characteristics of participants

Of 2,329 included in this study, 1,721 patients had a final clinical diagnosis of TB, and 608 participants were excluded from TB. The median age of the clinically diagnosed group was 56 (32, 68) years and the median age of the patients excluded from TB was 53 (33, 56) years. The majority of patients in the clinically diagnosed TB group were male (72.8%), with the youngest being 9 years old and the oldest being 96 years old, with the greatest number of older people over 65 years of age (35.3%), followed by those aged 45–64 years (28.7%). 96.0% of the specimens were sputum and the remaining 4.0% were BALF. 76.5% of the patients had not received systematic treatment for TB, with rural areas comprising the majority of the population (62.6%), among the different occupations, the highest number of patients was among the farmers and the unemployed, accounting for 32.7% and 34.3%, respectively. 1.2% of the patients were HIV-infected and 19.7% were diabetic (Table 1).

**Table 1:** Socio-demographic and clinical profiles of patients

Characteristics	TB n (%)	Non-TB n (%)
Gender		
Male	1,253(72.8)	404(66.4)
Female	468(27.2)	204(33.6)
Age categories/years		
Median (quartile)	56(32,68)	53(33,65)
< 14y	18(1.1)	7(1.1)
14-24y	222(12.9)	79(13.0)
25-44y	379(22.0)	135(22.2)
45-64y	494(28.7)	223(36.7)
> 65y	608(35.3)	164(27.0)
Specimen type		
Sputum	1,653(96.0)	569(93.6)
BALF	68(4.0)	39(6.4)
TB treatment history		
Primary treatment	1,316(76.5)	…
Rehabilitation	405(23.5)	…
Area		
Town	643(37.4)	226(37.2)
Countryside	1,078(62.6)	382(62.8)
Careers		
Farmer	562(32.7)	193(31.7)
Active Staff	169(9.8)	62(10.2)
Unemployed	591(34.3)	231(38.0)
Student	121(7.0)	53(8.7)
Others	278(16.2)	69(11.3)
AIDS	20(1.2)	68(11.2)
Diabetes	339(19.7)	50(8.2)

*BALF* Bronchoalveolar lavage fluid

### Positive rate of results of various assays

**Fig. 7:**
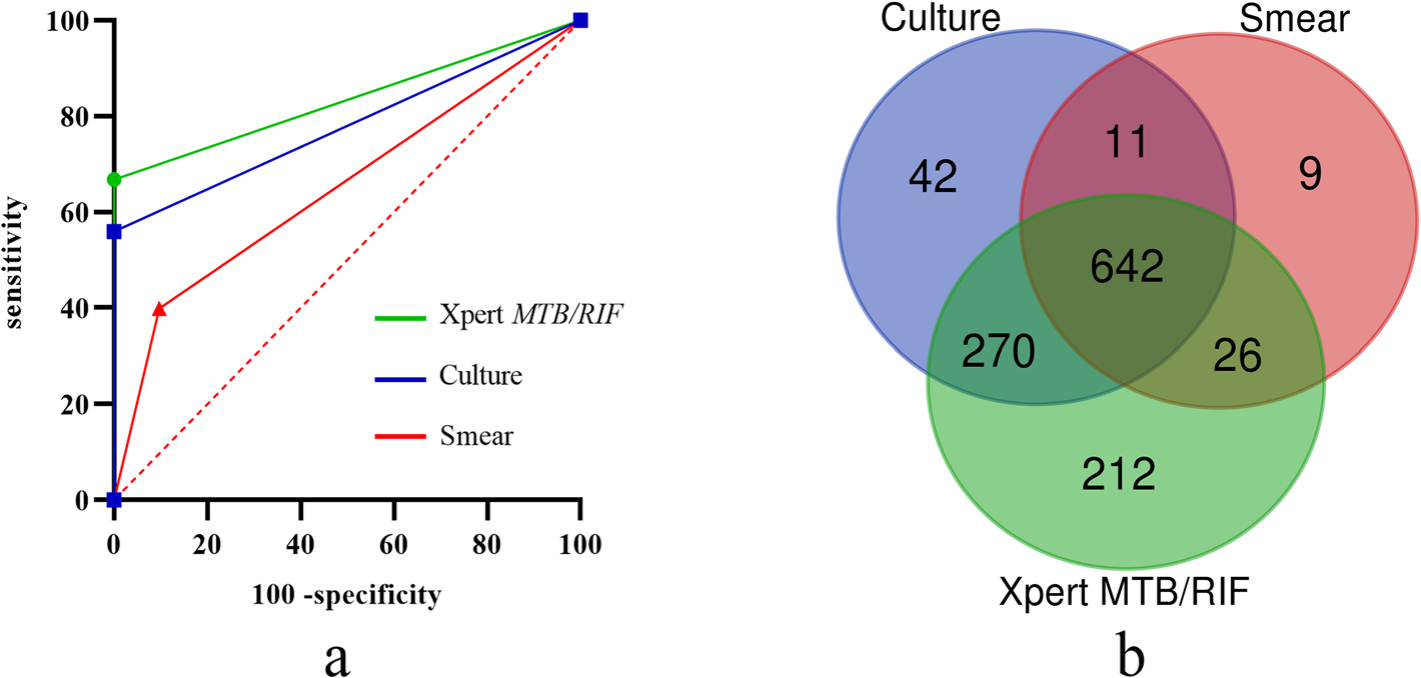
(**a**) Receiver operating characteristic (ROC) of the three methods for all cases. The area under the curve (AUC) value obtained for Xpert was 0.83, and AUC values of 0.78 and 0.65 for the culture and smear methods, respectively; (**b**) Venn diagram of positive *MTB* results of the three tests

Among 1,721 clinically diagnosed patients the results of the sputum smear method were positive in 688 (40.0%) cases, of which 1 + sputum specimens were the most in 288(16.7%) cases, and trace amounts were the least in 39 (2.3%) cases. The results of the traditional L-J culture method were positive in 965 (56.1%) cases, and 114 (6.6%) cases were identified as rifampicin-resistant by the drug sensitivity of the proportional method, with a resistance rate of 11.8%. The Xpert method had the highest detection rate of 1,150 (66.8%) cases, with the lowest number of very low cases at 193 (11.2%) and the highest number of moderate cases at 378 (22.0%). Among these, rifampicin-resistant strains were found in 133 (7.7%) cases, along with 14 (0.8%) cases that resulted in “indeterminate” results. Of the 608 cases in the non-TB group, 58 (9.5%) were positive by smear, and based on the identification results, all of these patients were eventually diagnosed with *NTM* infection (Table 2). ROC curves were generated for further analysis (Fig. 7a) and the AUC values of the ROC curves for the Xpert method were significantly higher than those of the culture (0.83 vs. 0.78, p < 0.001) and smear (0.83 vs. 0.65, p < 0.001). The distribution and overlap of the positive results obtained by the three assays are shown in Fig. 7b.

**Table 2:** Positive rate of results of various assays

Test Methods/Results	TB n (%)	Non-TB n (%)
Smear		
Positive	688(40.0)	58(9.5)^a^
Trace	39(2.3)	6(1.0)
1 +	288(16.7)	52(8.5)
2 +	124(7.2)	0
3 +	126(7.3)	0
4 +	111(6.5)	0
Culture		
Positive	965(56.1)	0
RR	114(6.6)	0
Xpert		
Positive	1,150(66.8)	0
Very low	193(11.2)	0
low	290(16.8)	0
Medium	378(22.0)	0
High	289(16.8)	0
RR	133(7.7)	0
Indeterminate	14(0.8)	0

*AFB* acid-fast bacilli, *RR* rifampicin resistance

^a^Identified as *nontuberculous mycobacteria*

### Comparison of culture and Xpert results at different AFB levels

We evaluated the diagnostic efficacy of the Xpert method using traditional L-J culture results as a reference standard. Among all 1,721 clinically diagnosed cases, the sensitivity and specificity of the Xpert method were 94.4% and 68.5%, respectively, the largest number of specimens were negative for smear, with 1033 cases, of which 313 specimens were positive for culture, while 482 specimens were positive for Xpert method results, whose sensitivity and specificity were 86.3% and 70.6%, respectively. In samples with high sputum smear load (AFB > 1 +), the sensitivity of Xpert was greater than 95% in all cases. The specificity was lower in all cases (Table 3).

**Table 3:** Comparison of L-J culture and Xpert results at different AFB levels

AFB	Number (n)	Sensitivity (%)	Specificity (%)	PPV (%)	NPV (%)
Negative	1,033	86.3	70.6	56.0	92.2
Trace	39	94.0	33.3	88.6	50.0
1 +	288	97.8	28.6	94.6	50.0
2 +	124	99.2	20.0	96.7	50.0
3 +	126	99.2	0	97.6	0
4 +	111	99.1	0	100	0
Total	1,721	94.4	68.5	79.3	90.5

*AFB* acid-fast bacilli, *PPV* Positive predictive value, *NPV* Negative predictive value

### Xpert results with different combinations of sputum smear and culture results

We evaluated the detection efficacy of the Xpert method by grouping different sputum smear results and culture results when the results of both methods were positive, the Xpert method had the highest detection rate of 98.3%, of which 88 (13.5%) were rifampicin-resistant, and the detection rate was the next highest at culture-positive + AFB-negative at 86.3%, of which 25 (8.0%) were rifampicin-resistant, followed by AFB-positive + culture-negative specimens with a detection rate of 74.3%. In contrast, 720 specimens were negative by both methods, of which 212 were still detected by the Xpert method for *MTB*, with a detection rate of 29.4%. RR was detected in 20 (2.8%) of the double-negative specimens (Table 4).

**Table 4:** Detection efficiency of the Xpert method at different AFB and L-J culture results

AFB	Culture	n	Xpert		Percentage(%)	Xpert method RR n (%)
			+	-		
+	+	653	642	11	98.3	88(13.5)
+	-	35	26	9	74.3	0(0)
-	+	313	270	43	86.3	25(8.0)
-	-	720	212	508	29.4	20(2.8)

*AFB* acid-fast bacilli, *RR* rifampicin resistance

### Comparison of RR between DST and Xpert methods

In this study, 1,150 specimens were positive by the Xpert method, of which 14 had “indeterminate” sensitivity results, which were firstly excluded, and of the remaining 1,136 specimens, 232 were negative by the L-J culture method, which was then excluded. Rifampicin resistance results were compared between Xpert and L-J culture methods in the remaining 904 patients. We also analyzed the consistency of their test results. The results showed a very high concordance (κ = 0.92) between the two methods for detecting RR in specimens with positive results from both L-J culture and Xpert methods. The level of the κ value did not vary with the load (Table 5).

**Table 5:** Comparison of rifampicin resistance between DST and Xpert methods

Xpert	n	Xpert	DST		Kappa
			R	S	
Very low	75	R	8	2	0.82
		S	1	64	
Low	200	R	20	5	0.79
		S	4	171	
Medium	350	R	36	0	0.99
		S	1	313	
High	279	R	39	3	0.98
		S	0	237	
Total	904	R	103	10	0.92
		S	6	785	

*DST* drug sensitization test, *R* resistant, *S* sensitivity

**Fig. 8:**
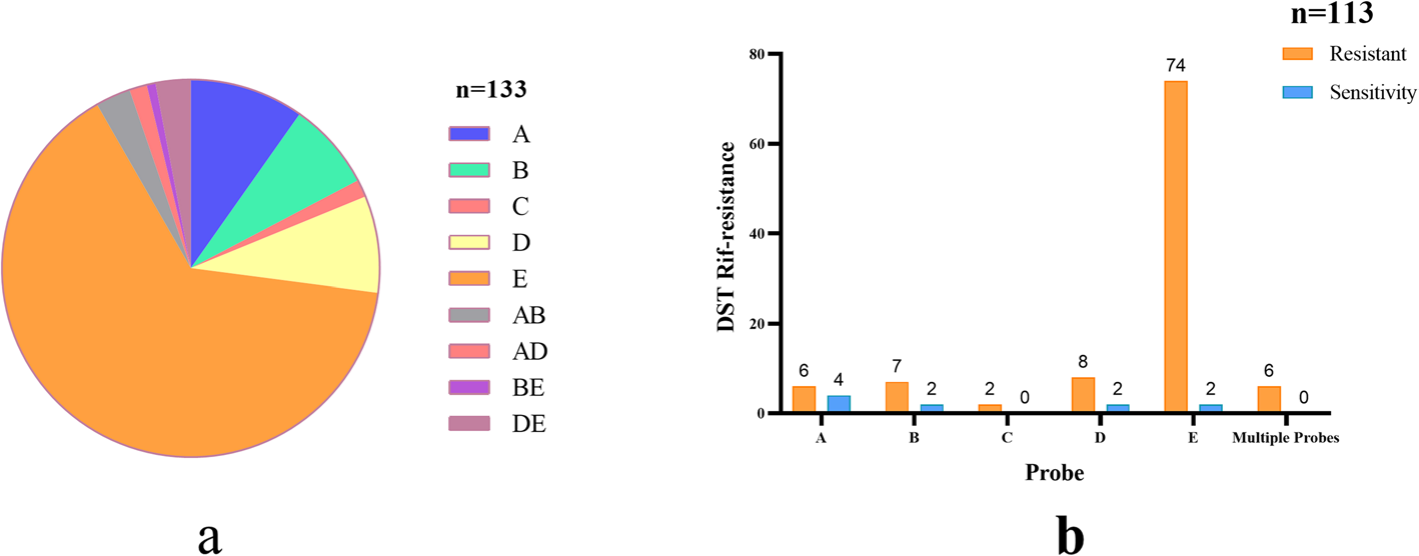
**a** Mutation sites in genotype-resistant samples by the Xpert method. **b** Phenotypic resistance upon mutation of various probes

**Fig. 9:**
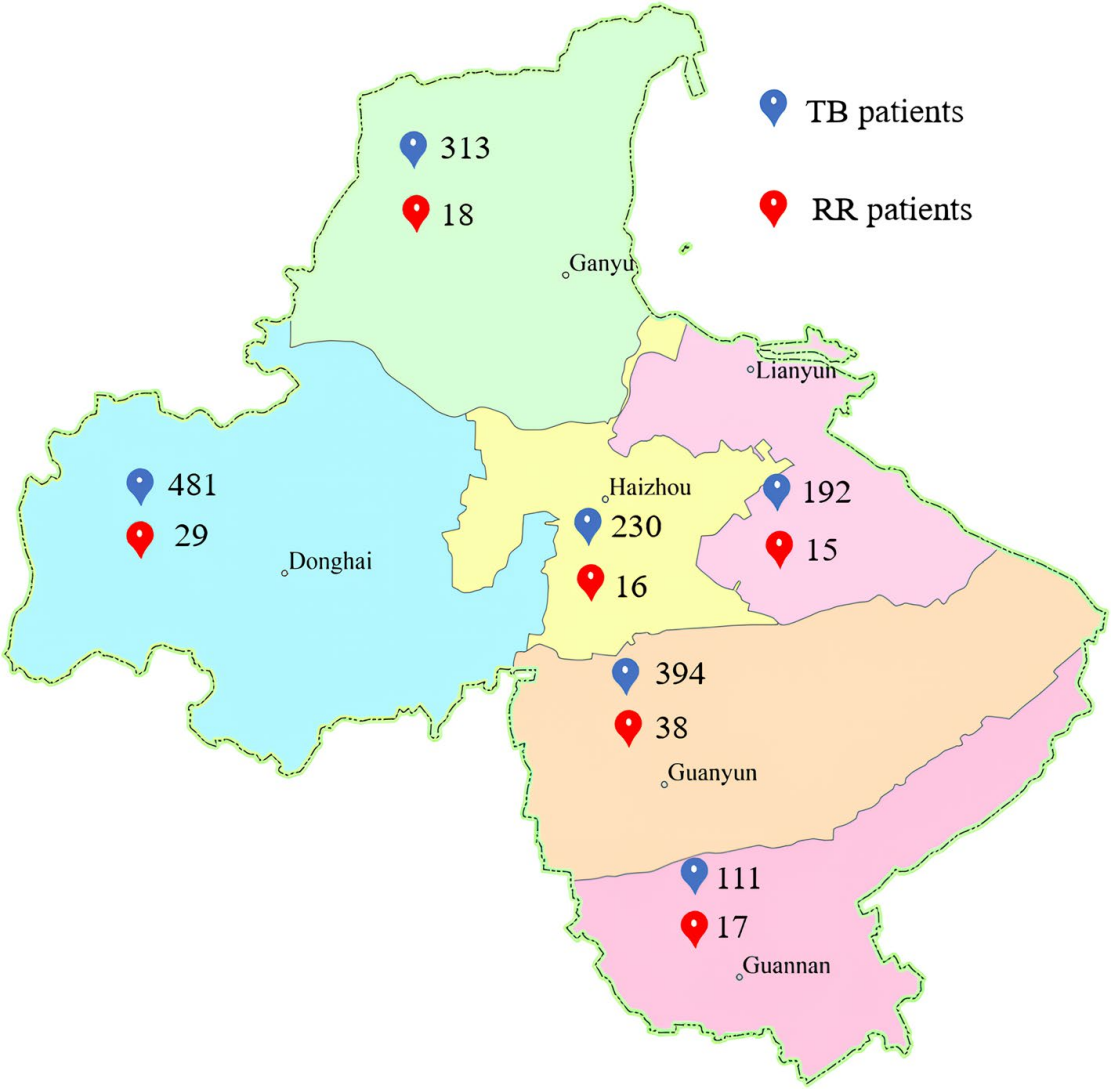
Geographic distribution of patients

Our further analysis of the phenotypes and genotypes of the drug sensitivity experiments, Xpert results for RR in 133 specimens, of which the largest number of probe E mutations (86), followed by probe A (13), probe B (10), probe D (11), which were not significantly different from each other, and the number of probe C mutations was 2, and the rest of the mutations were multiple probe mutations (11) (Fig. 8a). While 113 of these 133 specimens were positive by culture, 10 of them had rifampicin-sensitive DST results. We refined them and found that the lowest resistance rate of DST was 60.0% when probe A was mutated. The resistance rate of DST was 77.8% when probe B was mutated, 80.0% when probe D was mutated, and 97.4% when probe E was mutated, whereas the results of DST were resistant when the probe C was mutated and when multiple probes were mutated (Fig. 8b). The geographic distribution of clinically diagnosed TB patients and rifampicin-resistant patients detected by the Xpert method in this study is shown in Fig. 9.

## Discussion

Diagnosis of tuberculosis (TB) has always been a major global public health challenge. Rapid and accurate laboratory diagnosis and the development of rational treatment regimens are critical to preventing and controlling TB [26]. However, it has been difficult to effectively control TB because of the atypical clinical symptoms of early TB and the insidious nature of the lesions [27]. Timely and accurate diagnosis of AFB-negative TB, in particular, is challenging. Based on microscopic detection techniques, the smear method remains the first-line test in most resource-poor endemic areas [10, 18, 28]. It has been reported that the detection rate of TB by the smear method is only 12.6% of the number of TB transmissions [7, 29], and due to its low sensitivity, smear microscopy misses many cases of TB, resulting in patients not being diagnosed and treated promptly [30]. Strain identification and DST based on traditional culture methods are considered the gold standard for diagnosing TB, but the process is time-consuming, taking weeks or even months [31]. In addition, *MTB* culture and DST require extensive and expensive equipment, which poses a challenge in endemic areas with limited resources or low economic levels [25]. The Xpert method has excellent sensitivity and specificity in detecting *MTB* infections and RR and has a short detection time [32]. In this study, we compared the diagnostic results of smear, culture, and Xpert method between June 2020 and December 2023 in the Fourth People's Hospital of Lianyungang City.

A total of 1,721 cases of clinically diagnosed TB were included in this study, of which males (72.8%) were more than twice as many patients as females (27.2%), which is consistent with the findings of several studies [33, 34]. In a report from Ethiopia, it was noted that the male gender is an established risk factor for TB [35]. The high prevalence of TB among men in low- and middle-income countries may be attributed to poor health-seeking behavior and lack of active engagement in TB treatment [36], that men are less likely to receive a timely diagnosis of TB, men are less health-conscious relative to female patients, and that male patients are more likely to delay seeking medical care, and therefore men are at relatively higher risk of developing TB, and disadvantages in accessing TB diagnosis and treatment suggest that undiagnosed TB cases are higher among men [37]. Previous reports have shown that *MTB* drug resistance is more likely to occur in low-income populations [34, 36]. Among the cases in the current study, patients in Guanyun and Guannan counties, which have the lowest economic levels, had the highest rates of drug resistance, 9.6%, and 15.3%, respectively. In the grouping by age, the highest number was found in the older age group of 65 years and above. This is inconsistent with the results reported by Christine Vanlalbiakdiki Sailo, Kwame Kumi Asare, Getu Diriba, et al. [5, 10, 24], whose study considered younger males to have the highest number of TB infections. However, our findings are consistent with those of several other studies. Their research suggests that aging-related biological changes, decreased cellular immune responsiveness, acute and chronic illnesses, poor nutrition, and reduced access to timely healthcare resources may contribute to the higher incidence of tuberculosis in the elderly [38, 39].

TB cannot be ruled out solely based on a negative result from a single test method; the underdiagnosis of TB cases can lead to serious negative consequences, such as progression to severe extrapulmonary TB, TB encephalitis, organ failure, and death, especially in immunocompromised populations, including children, the elderly, and individuals living with HIV [40, 41]. In addition, patients with AFB-negative TB can still be a source of infection for TB transmission [42]. A study by E Hernández-Garduño et al. states that at least 17.3% to 41% of TB cases are transmitted through AFB-negative TB patients [43]. The diagnosis of TB based on microscopic testing has several drawbacks, including low sensitivity and specificity, a minimum limit of detection (LOD) of 5,000 to 10,000 CFU/ml for the smear method, the dependence of smear microscopy on bacillus antacids that are visible under the microscope, which may miss cases with low loads of or invisible bacilli, and the inability to differentiate between *MTB* and *NTM*, which are drawbacks that may result in a high rate of missed diagnosis and false positives [44]. In contrast, the Xpert method targets the DNA of *MTB* in the sample, which gives the Xpert method greater sensitivity and the ability to detect low loads and AFB-negative samples [45]. Among the clinically diagnosed cases in this study, sputum smear had the lowest positive rate of 40.0% (688/1,721). The L-J culture method had a positive rate of 56.0% (965/1,721) and the Xpert method had the highest positive rate of 66.8% (1,150/1,721). This is consistent with previous reports [10, 46, 47].

Before genetic diagnostic techniques were widely used in clinical laboratories, most laboratories relied on methods based on clinical presentation, smear, and L-J culture methods, supplemented by immunology and imaging to diagnose tuberculosis [48]. However, due to problems of sensitivity and detection thresholds, relying on these methods alone resulted in a high rate of missed detections [23]. Our study confirms that the Xpert method can effectively fill these gaps. When we grouped the cases based on the results of sputum smear and L-J culture methods, we found that the Xpert method had a lower leakage rate than the other two methods. When both methods were positive, the detection rate of the Xpert method was 98.3% (642/653), and of the 348 cases in which only one of the two methods was positive, the Xpert method detected 296 of them, a detection rate of 85.1% (296/348). It is challenging to diagnose patients with symptoms of TB who have negative smears and cultures because of the lack of etiological support, which complicates medication use and diagnosis in the clinic [49]. The Xpert method detection rate of 29.4% (212/720) of such samples in the present study alleviates this issue. In addition, 20 of the 212 samples detected by the Xpert method were rifampicin-resistant. This proves that the Xpert method also has a positive significance in minimizing the rate of underdetection of rifampicin-resistant tuberculosis.

We are concerned about the difference in results between the Xpert and culture methods. In the present study, among the specimens that were positive by the Xpert method, the culture method was positive in 79.3% (912/1,150). Among the cases with negative sputum smear results, this percentage was 56.0% (270/482). In specimens with positive sputum smear, it was 96.1% (642/668). When we compared the detection efficacy of the Xpert method using the results of the culture method as a reference standard, we found that the Xpert method had a very high sensitivity and a low specificity, a result that is consistent with the findings of Willy Ssengooba, Emmanuel Musisi, Juan Yang et al. [13, 14, 50]. In the total number of specimens, the sensitivity and specificity of the Xpert method were 94.4% and 68.5%, respectively. In AFB-negative specimens, the sensitivity and specificity were 86.3% and 70.6%, respectively. In AFB-positive specimens, they were 98.3% and 25.7%, respectively. The low concordance between the results of the Xpert method and those of the culture method may be attributed to several factors. Firstly, the traditional solid culture method is not a perfect “gold standard”, and some true-positive cases may be missed due to the quality of samples, manipulation practices, reagent configuration, culture environment, and other factors [51]. The process of sample processing, including sample separation, selection, and addition of NaOH solution, may cause the *MTB* concentration in the sample to decrease or die, resulting in a decrease in the sensitivity of the culture [33]. In addition, we know that the Xpert method detects the DNA of *MTB* in the sample, and even if the *MTB* in the specimen is dead, the Xpert method can still yield a positive result. Therefore, further workup through multiple sampling, follow-up studies, and other complementary tests including imaging is essential in Xpert-positive and culture-negative cases. On the other hand, we found that some patients with L-J culture or AFB-positive results and negative Xpert methods persisted after excluding *NTM* infections, and this phenomenon tended to occur in samples with low loads. We hypothesize that factors contributing to this phenomenon include the possible presence of PCR amplification inhibitors such as bile acid salts, bilirubin, and hemoglobin in the samples. In these cases, multiple, standardized retention of samples and repeated testing should be used to confirm the accuracy of the results.

In conjunction with the detection of *MTB*, the Xpert method can also provide RR results by detecting mutations in the *rpoB* gene [25]. In this study, 133 cases were identified as genotypically resistant by the Xpert method, and 113 instances exhibited phenotypic resistance as determined by the proportional method drug sensitivity test. Among the genotypically resistant cases, the E probe mutation was the most prevalent at 64.6%, followed by A (9.8%), D (8.3%), B (7.5%), and C (1.5%). The remaining 8.3% consisted of multiple probe variants. This result is consistent with our previous findings [52]. There is a discrepancy between our results and those Christine Vanlalbiakdiki Sailo et al. reported. [24], in whose study the most mutations were found in probe A, followed by E, D, B, and C. However, our study is consistent with most reports, with E probe mutations being the most prevalent, as stated in Ayinalem Alemu's and Irfan Ullah's studies [53, 54]. These discrepancies may be due to geographic differences in the spectrum of *MTB*.

We evaluated the value of the Xpert method for rifampicin drug sensitivity experiments using the DST drug sensitivity results as a reference standard after excluding negative results. We found excellent agreement (κ > 0.75) between the Xpert method and the DST drug sensitivity results regardless of the TB load in the samples. The drug sensitivity results of the two were inconsistent in 16 samples. Previous studies have shown that about 95% of RR is associated with mutations in the 81 bp hotspot region of the *rpoB* gene [24], and in our current study, 5.5% (6/109) of these samples had a DST result of resistance, while the Xpert method showed a sensitivity result. This is in line with previous studies. An additional 10 samples exhibited phenotypic results of sensitivity but genotypic results indicating resistance. *rpoB* gene mutations may lead to a complementation degree of RR depending on the location of the mutation and the protein changes caused by the mutation [55]. In the current study, 40.0% (4/10) of the samples had probe A mutations, 22.2% (2/9) of probe B mutations, 20.0% (2/10) of probe D mutations, and 2.6% (2/76) of probe E mutations had sensitive DST results. Previous studies have shown that mutations occurring in probes D and E result in high levels of RR, while mutations in probes A and B were associated with low levels of RR. Amino acid changes resulting from multiple mutation types at loci 511, 516, 526, and 533 have been reported to be controversial mutation types, and they may result in genotypic resistance but phenotypic sensitivity [55–57]. Therefore, due to such phenomena, we believe it is not sufficient to fully determine RR in strains based solely on the genetic mutations detected in the Xpert method and that the DST method remains important and irreplaceable.

However, there are also limitations to this work. First, only 10 patients with TB had extrapulmonary specimens during the time frame of this study. Therefore, this study did not explore the detection efficacy of the Xpert method for specimens from sites outside the lungs. Second, the sputum specimens are collected multiple times and retained in several copies; some patients may have variations in the quality of specimens collected at different times, leading to biased test results. To address this issue, at the time of specimen collection, we asked patients with poorer quality specimens to re-collect their specimens to minimize the impact caused by inter-bottle variation.

## Conclusions

The Xpert method has good diagnostic value in detecting *MTB* and rifampicin resistance and has shown good efficacy in cases where conventional culture and sputum smear tests were negative. This has a positive effect on early diagnosis and treatment of TB before the results of traditional culture and drug susceptibility testing are available. However, caution should be exercised with positive Xpert assay results, and a combination of multiple test results and clinical evaluations is necessary to determine the patient's final diagnosis and treatment plan.

## Data Availability

Data underlying the results presented in this paper are not publicly available at this time but may be obtained from the authors upon reasonable request.
